# The Solubility of Diphtheritic Membranes

**Published:** 1868-12

**Authors:** 


The Solubility of Diphtheritic Membranes.—MM. Brichetau and Adrian (*Union Pharm.*) have made a number of experiments to test the solubility of "false membrane" in various medicinal substance; and announce, among other things, the following results:

A false membrane maintained for an hour in the midst of vapors of sulphate of mercury is not dissolved; it is only softened as by vapor of water; retained in a concentrated solution of pepsin maintained at a temperature of 30° C., it is not dissolved but at the end of 12 hours; but if 6 to 10 drops of lactic acid be added to the pepsin, the solution is accomplished at the end of 8 minutes. Caustic acids (hydrochloric, sulphuric, azotic) do not dissolve false membranes. Acetic acid renders the diphtheritic membrane transparent, gelatinous, but does not dissolve it completely. Citric acid produces a similar effect. Lactic acid, in the proportion of 2 drops to 5 grammes of water, dissolved a tough membrane of the weight of 20 centigrammes in 3 minutes. With limewater, the effect is still more rapid; lactic of lime is without action. Water alkalinized by soda or potassa, dissolves the membrane in 8 or 10 minutes, and better than their concentrated solu:ions. Bromine water, and bromine in statu nascente, only disintegrate the membrane; bromide of potassium has no apparent actiont salts of soda and potassa, such as sulphate of soda, sulphate of potassa, the bicarbonate, nitrate, etc., are without action, as also chloride of zinc, and chromic acid. The chlorates of potassa and soda dissolve membranes, but slowly.—*L' Univ. Med.*

